# miR-129-5p inhibits clear cell renal cell carcinoma cell proliferation, migration and invasion by targeting SPN

**DOI:** 10.1186/s12935-021-01820-3

**Published:** 2021-05-17

**Authors:** Bin Gao, Lijuan Wang, Na Zhang, Miaomiao Han, Yubo Zhang, Huancai Liu, Dongli Sun, Xiaolong Xiao, Yifei Liu

**Affiliations:** 1 Department of Urology, Tangshan Central Hospital, East of Guangming Road, South of Longfu South Road, West of Youyi Road West Auxiliary Road, North of Changning Road, Tangshan, 063000 China; 2 Shanghai Engineering Research Center of Pharmaceutical Translation, Shanghai, China

**Keywords:** SPN, miR-129-5p, Clear cell renal cell carcinoma, Proliferation, Migration and invasion, Apoptosis

## Abstract

**Objective:**

Our study aims to investigate the mechanism of the miR-129-5p/SPN axis in clear cell renal cell carcinoma (ccRCC), providing a novel direction for the targeted therapy of ccRCC.

**Methods:**

Bioinformatics methods were implemented to find the differentially expressed genes (DEGs) associated with ccRCC from TCGA database. qRT-PCR was performed to detect miR-129-5p and SPN mRNA expression, while western bot was carried out for the detection of protein expression of SPN. Bioinformatics analysis was used to predict the binding sites of miR-129-5p on SPN 3’UTR, while dual-luciferase assay was conducted to verify their binding relationship. CCK-8 assay, colony formation assay, wound healing assay and Transwell assay were employed to measure ccRCC cell proliferative ability, cell formation ability, cell migratory and invasive abilities. Flow cytometry was implemented to assess cell cycle and apoptosis.

**Results:**

miR-129-5p exhibited a significantly down-regulated expression level in ccRCC, while SPN showed a remarkably up-regulated expression level. Overexpressed miR-129-5p inhibited ccRCC cell proliferative, invasive and migratory capacities while induced cell cycle arrest in G0/G1 phase and promoted cell apoptosis. Dual-luciferase assay confirmed that there was a binding relationship between miR-129-5p and SPN. Moreover, overexpressed miR-129-5p remarkably reduced SPN expression in cancer cells, weakened the promoting effect of SPN on cell proliferation, migration, invasion and cell cycle progress, and led to enhanced cell apoptotic activity.

**Conclusions:**

Our study proves the regulatory effect of the miR-129-5p/SPN axis in ccRCC, and provides a novel potential target for precise treatment of patients with ccRCC.

## Introduction

Renal cell carcinoma (RCC), accounting for 3 % of all adult cancers, is the most common malignancy in adult and one of the leading causes of cancer-related deaths worldwide, with a mortality rate of over 40 % [1]. Clear cell renal cell carcinoma (ccRCC) is the most common subtype of RCC and is predicted to take up 75–80 % of RCC tumors [2]. Although enormous progress has been made towards the treatment of cancer, the prognosis of patients with locally advanced and metastatic RCC remains unsatisfactory [3], and the 5-year survival rate is still low. It has been reported that up to 30–40 % of patients with RCC still have metastatic diseases even after the radical resection [4]. Therefore, it is necessary to enrich our knowledge about the molecular mechanism that regulate the metastatic and invasive abilities of ccRCC and study the potential mechanisms underlying ccRCC, so as to develop a novel therapeutic method.

MicroRNAs (miRNAs) are endogenous small non-coding RNAs that are emerging as a novel tool for regulating gene expression. A study has indicated that miRNAs are involved in regulating various biological events, such as cell proliferation, differentiation and apoptosis [5]. Therefore, miRNAs are related to the occurrence of several human diseases, including tumorigenesis [6]. A research suggests that the dysregulation of miRNAs is associated with the biological characteristics of ccRCC, and it may serve as a novel potential biomarker for prognosis and treatment [7]. miR-129-5p is a key tumor regulatory factor that plays an important role in the progression of cancers. Numerous studies have elucidated that miR-129-5p is considerably differentially expressed and exerts an essential role in colon cancer [8], liver cancer [9], pancreatic cancer [10 and gastric cancer, etc. [11]. Despite that the regulatory role of miR-129-5p in ccRCC has been studied before [12], the regulatory mechanism of miR-129-5p in the occurrence and development of ccRCC requires further exploration.

Sialophorin (SPN) also known as LSN and CD43 according to NCBI (https://www.ncbi.nlm.nih.gov/gene/6693) is a transmembrane saliva glycoprotein. In human, in addition to be present in mature red blood cells and B cell subsets, SPN has also been found to be expressed in hematopoietic cells, including T lymphocytes, monocytes, granulocytes, natural killer cells, platelets, and hematopoietic stem cells [13]. Generally speaking, SPN has the capacity of regulating intercellular adhesion, intracellular signal transduction, cell apoptosis, cell migration and proliferation [14]. There are few studies regarding the regulatory role of SPN in cancers, and SPN has only been reported to be a new therapeutic target in breast cancer [14]. Besides, the relevance between the dysregulation of SPN and the occurrence of ccRCC is uncharacteristic. Hence, exploring the regulatory role of SPN in ccRCC will provide a theoretical basis for SPN acting as a cancer regulatory factor.

In this study, we predicted the target gene SPN of miR-129-5p by bioinformatics analysis. Our study aims to elucidate the effect of the miR-129-5p/SPN axis on the occurrence and development of ccRCC, providing a theoretical basis for exploring novel biomarkers and targeted therapies for ccRCC.

## Materials and methods

### Bioinformatics analysis

TCGA is currently the largest cancer genetic information database. ccRCC miRNA-seq (normal: 71, tumor: 536) and mRNA-seq (normal: 72, tumor: 530) expression data were downloaded from TCGA database (https://portal.gdc.cancer.gov/). “edgeR” package was employed to obtain the differentially expressed miRNAs and mRNAs (DEmiRNAs, DEmRNAs) with threshold set as |logFC|>2 and FDR < 0.05, while “survival” package was used for survival analysis to identify the target DEmiRNA. miRDIP (http://ophid.utoronto.ca/mirDIP/index.jsp#r) and miRDB (http://mirdb.org/) databases were applied to conduct target prediction for the miRNA. The mRNA with binding sites of the miRNA was identified from the intersection of DEmRNAs in TCGA database and predicted genes.

### Cell culture

Human renal tubular cell line HK-2 (BNCC339833), human renal carcinoma cell line A498 (BNCC100609), and human ccRCC cell lines 786-O (BNCC338472), 769-P (BNCC100976) and Caki-1 (BNCC100682) were all purchased from BeNa Culture Collection (BNCC; China). All cell lines were cultured in Dulbecco’s Modified Eagle’s Medium (DMEM; BNCC351841, BNCC, China) containing 10 % fetal bovine serum (FBS), and maintained in an incubator with 5 % CO_2_ at 37 ℃.

### Cell transfection

miR-129-5p mimics (miR-mimics), mimics NC (miR-NC), SPN overexpression vector pcDNA3.1-SPN (oe-SPN) and empty vector pcDNA3.1 for negative control (oe-NC) accessed from Ribobio (China) were transfected into ccRCC cell line 786-O by Lipofectamine 2000 kit (Invitrogen, Carlsbad, USA) in accordance with instructions. After 24 h of transfection, transfected cells were used for subsequent experiments.

### Real‐time fluorescence quantitative PCR

Total RNA was isolated from cells using TRIzol kit (Life Technologies, USA), and its concentration was measured by NanoDrop 2000 system (Thermo Fisher Scientific, Inc., USA). miRNA was reversely transcribed into cDNA by miScript II RT kit (Qiagen, USA), while mRNA was transcribed into cDNA by PrimeScript RT Master Mix (Takara, P.R. China). miRNA expression and mRNA expression were detected by miScript SYBR Green PCR Kit (Qiagen, Germany) and SYBR ® Premix Ex Taq TM II (Takara Bio Inc., Japan), respectively. qRT-PCR was performed to assess miR-129-5p and SPN mRNA expression through Applied Biosystems® 7500 Real-Time PCR Systems (Thermo Fisher Scientific, MA) with U6 and GAPDH taken as internal reference, respectively. Primer sequences used in qRT-PCR were listed in Table 1. The relative expression of miR-129-5p and SPN mRNA was presented by 2^−ΔΔCt^ method. The experiment was repeated three times.

**Table 1:** Primer sequences used in qRT-PCR

Gene	Primer sequence	
miR-129-5p	Forward	5′-GGGGGTTTTTGCGGTCTGG-3′
	Reverse	5′-AGTGCGTGTCGTGGAGTC-3′
U6	Forward	5′-CTCGCTTCGGCAGCACA-3′
	Reverse	5′-AACGCTTCACGAATTTGCGT-3′
SPN	Forward	5′-GCAAACUCUCUAGG AUCCCTT-3′
	Reverse	5′-GGGAUCCUAGAGAGUUUGCTG-3′
GAPDH	Forward	5′-GAAGGTGAAGGTCGGAGTC-3′
	Reverse	5′-GAAGATGGTGATGGGATTTC-3′

### Western blot

Total proteins were harvested after cells were lysed by RIPA lysis buffer, and protein concentration was assayed by BCA kit (Beyotime, China). After being denatured at a high temperature, proteins were isolated by sodium dodecyl sulfate-polyacrylamide gelelectrophoresis (SDS-PAGE) and transferred onto polyvinylidene fluoride membranes (PVDF; Millipore), which were then blocked with 5 % skim milk for 2 h. Then the membranes were incubated with primary antibodies overnight at 4 ℃. Primary antibodies including mouse anti-SPN (ab233969, 1:100) and mouse anti-β-Actin (ab20272, 1:5000) were both purchased from Abcam (Shanghai, China). Subsequently, the membranes were incubated with secondary antibody goat anti-mouse IgG (ab205719; abcam, Shanghai, China). After culture for 2 h at room temperature, all protein bands were visualized using an enhanced chemiluminescence kit (GE Healthcare, USA). The experiment was conducted in triplicate, and original western blot gel image were provided as Additional file 1.

### Cell proliferation assay

CCK-8 assay was applied for the detection of cell proliferative ability. Cells were seeded into 96-well plates at a density of 2 × 10^4^ cells/well and incubated with 5 % CO_2_ at 37 ℃. 10 µl CCK-8 solution (CK04; Dojindo Laboratories, Japan) was added to each well at 0 h, 24 h, 48 h, 72 h and 96 h, followed by incubation for 2 h with 5 % CO_2_ at 37 ℃. The absorbance at 450 nm was measured by Microplate reader (Multiskan MK3, Thermo Fisher Scientific Inc., MA). The experiment was repeated three times.

### Colony formation assay

Colony formation assay was employed for the determination of colony forming ability. Transfected cells were planted into 6-well plates with a density of 1 × 10^3^ cells/well, and each procedure was run in triplicate. Transfected cells were then incubated in complete mediums for 14 days until the colonies were visualized to naked eyes. Cell colonies were fixed in 4 % paraformaldehyde for 15 min at room temperature and then stained by 0.05 % crystal violet (Thermo Fisher, USA) for 20 min. Sterile water was used to wash away the crystal violet in each well. Colonies (over 50 cells) were calculated in each well. The experiment was performed three times.

### Transwell invasion assay

24-well Transwell chambers (8 µm in aperture, BD Biosciences) were used for Transwell invasion assay. Approximately 2 × 10^4^ cells were placed into the upper chambers coated with Matrigel matrix, while the lower chambers were filled with DMEM supplemented with 10 % FBS. After being cultured for 48 h at 37 ℃, the non-invaded cells were removed through a wet swab cotton, while the invaded cells were stained with 0.1 % crystal violet. The images of cells were captured under the inverted microscope (DSX510i, Olympus, Japan), and five fields were randomly selected to count the invaded cells. The experiment was conducted three times.

### Wound healing assay

Wound healing assay was carried out to detect cell migratory ability. Cells were placed into 6-well plates. When cells grew to 80 % confluence, cell monolayers were wounded using a 200 µl pipette tip. Mediums were used to wash the cells twice to remove the isolated cells. Then, the cells remained were cultured in fresh medium for another 24 h. Cell migration was observed and images at 0 h and 24 h were caught. The experiment was repeated three times.

### Flow cytometry

For cell cycle analysis, cells of each group were pre-digested with 0.25 % trypsin and then washed in PBS twice. Following that, the cells were resuspended in prepared sample buffer which contained 20 µl propidium iodide (PI) (5 µg/ml) and 50 µl RNase A (10 mg/ml). Cell cycle was analyzed after 10 min of incubation away from light at 37 ℃.

For cell apoptosis evaluation, cells were resuspended by a binding buffer (pH 7.4) composed of 100 mmol/L 4-(2-hydroxyethyl)-1-piperazineethanesulfonic acid (HEPES), 100 mmol/L NaCl and 25 mmol/L CaCl_2_. Subsequently, the cells were washed with PBS twice, followed by 15 min of staining with Annexin V-fluorescein isothiocyanate (FITC)/PI in the dark at 37 ℃. Flow cytometry (BD Biosciences) was implemented to assess cell apoptosis. Each experiment above was repeated three times.

###  Dual‐luciferase assay

To determine the binding relationship between miR-129-5p and SPN 3′-UTR, luciferase vectors pmirGLO (Promega, USA) fused with wild type (WT) SPN 3’-UTR or mutant (MUT) SPN 3′-UTR were established. ccRCC cells 786-O were seeded into 96-well plates (3 × 10^5^ cells/well), and 100 nM miR-mimics/miR-NC and SPN-WT/SPN-MUT were co-transfected into cells. After culture for 48 h, luciferase activity was measured by dual-luciferase reporter assay system (Promega, USA). The experiment was performed in triplicate.

### Statistical analysis

All data were analyzed by GraphPad Prism 6.0 (La Jolla,CA), and the results were expressed in mean ± standard deviation. The comparison between two groups was analyzed by *t* test. * means *p* < 0.05, and *p* < 0.05 was considered statistically significant.

## Results

### miR-129-5p is down‐regulated in ccRCC cells

**Fig. 1:**
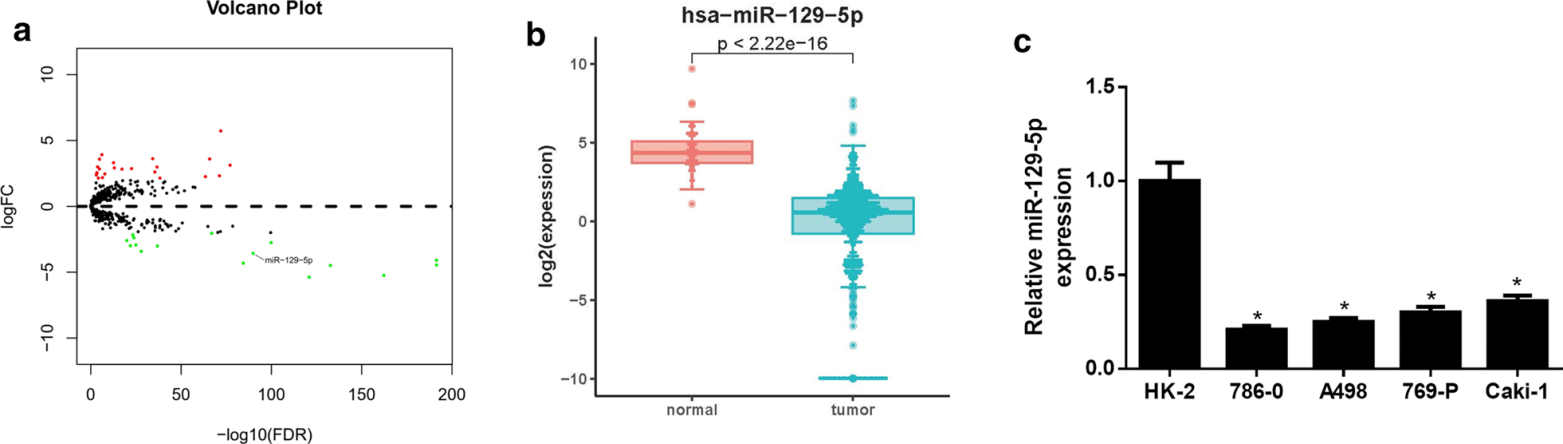
miR-129-5p is down-regulated in ccRCC cells. **a** Differential analysis of miRNA expression in the TCGA-KIRC dataset; **b** Box plots of miR-129-5p expression in normal group (n = 71) and tumor group (n = 536) in TCGA-KIRC dataset; **c** miR-129-5p expression in normal cell line HK-2 and ccRCC cell lines 786-O, A498, 769-P and Caki-1 was measured by qRT-PCR; * *p* < 0.05

39 DEmiRNAs were obtained by bioinformatics analysis on miRNA-seq expression data from TCGA database, among which 23 were up-regulated and 16 were down-regulated (Fig. 1a, Additional file 2). Several studies have revealed that miR-129-5p acts as a tumor suppressor in a variety of cancers. Hence, miR-129-5p was taken as the objective of this study. TCGA data showed that miR-129-5p was prominently down-regulated in ccRCC tissue (normal: 71, tumor: 536) (Fig. 1b). qRT-PCR was then employed to evaluate miR-129-5p expression in ccRCC cells and normal cells, finding that miR-129-5p was potently down-regulated in ccRCC cell lines (Fig. 1c). Collectively, these findings evinced that miR-129-5p was lowly expressed in ccRCC.

### Overexpressed miR-129-5p affects ccRCC cell biological functions

**Fig. 2:**
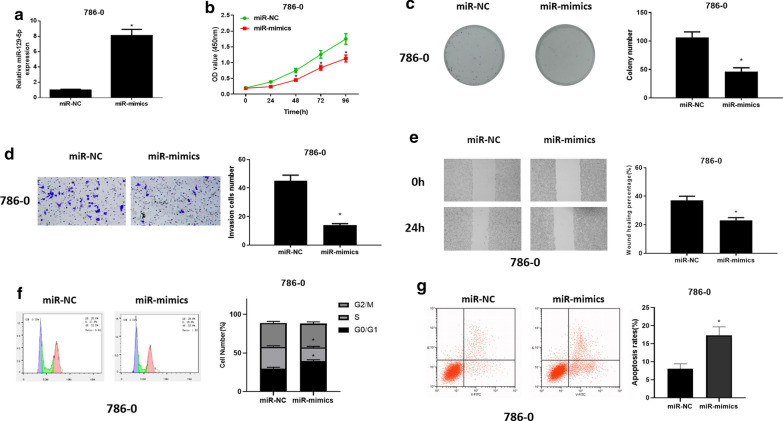
Overexpression of miR-129-5p affects ccRCC cell biological functions. **a** miR-129-5p expression was detected by qRT-PCR after miR-129-5p was overexpressed in ccRCC cells; **b** Cell proliferative ability, **c** colony forming ability, **d** cell invasive and **e** migratory abilities in different groups were measured by CCK-8 assay, colony formation assay, Transwell assay (100×) and wound healing assay (40×); **f** Cell cycle analyzed via flow cytometry; **g** Cell apoptosis evaluated by flow cytometry; * *p* < 0.05

A great number of researches have shown that the occurrence and development of tumors are related to the dysregulation of miRNA. miR-129-5p was found to be highly significantly abnormally expressed in ccRCC cell line 786-O. Therefore, ccRCC cell line 786-O was chosen for subsequent experiments. In order to investigate the biological function of miR-129-5p in ccRCC, miR-129-5p was overexpressed in ccRCC cell line 786-O. qRT-PCR was used to assess miR-129-5p expression in different groups, showing that miR-129-5p was remarkably up-regulated after miR-129-5p was overexpressed (Fig. 2a). CCK-8 assay, colony formation assay, Transwell assay and wound healing assay were carried out for detecting cell proliferation, colony forming ability, cell migration and invasion, finding that these abilities were greatly reduced after miR-129-5p was overexpressed relative to those of the control group (Fig. 2b–e). Additionally, findings in flow cytometry assays uncovered that upon the overexpression of miR-129-5p, the proportion of cells was increased in G0/G1 phase while decreased in S phase, and cell apoptotic rate was elevated (Fig. 2f, g). Taken together, these results demonstrated that overexpressed miR-129-5p suppressed ccRCC cell proliferation, migration and invasion, induced cell cycle arrest in G0/G1 phase and potentiated cell apoptosis.

###  SPN is potently up‐regulated in ccRCC cells

**Fig. 3:**
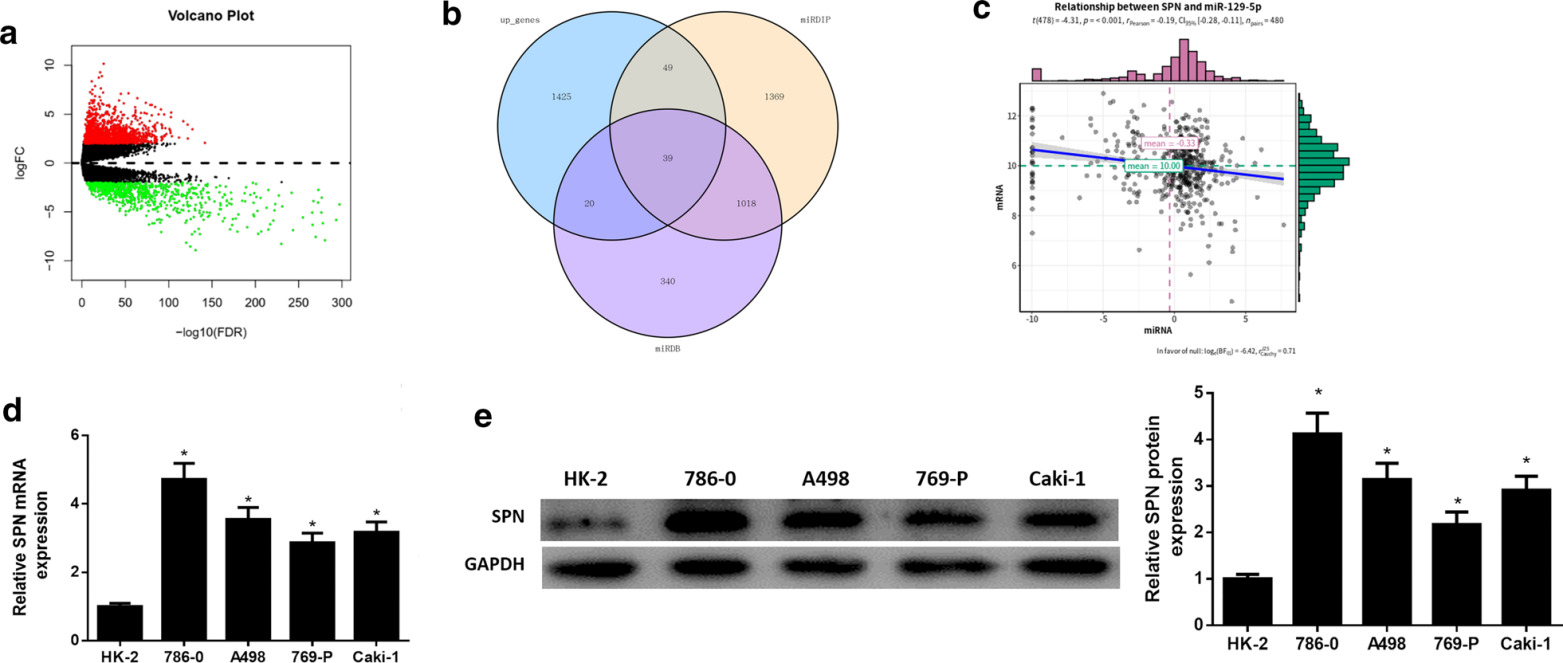
SPN is up-regulated in ccRCC. **a** Differential analysis of mRNAs in TCGA-KIRC dataset; **b** Venn diagram of predicted genes of miR-129-5p and up-regulated DEmRNAs in TCGA-KIRC dataset; **c** Pearson correlation analysis of miR-129-5p and SPN; **d** The mRNA expression and **e** protein expression of SPN in one normal cell line and five ccRCC cell lines were evaluated by qRT-PCR and western blot; * *p* < 0.05

To explore the regulatory mechanism of miR-129-5p in ccRCC cells, 2,312 DEmRNAs were obtained in TCGA-KIRC dataset by differential analysis, among which 1,533 were up-regulated and 779 were down-regulated (Fig. 3a, Additionl file 3). miRDIP and miRDB databases were employed to predict target genes of miR-129-5p, and then 39 candidate genes were obtained from the intersection of predicted genes and up-regulated DEmRNAs (Fig. 3b). Correlation analysis showed that miR-129-5p had the highest negative correlation coefficient (-0.19) with SPN (Fig. 3c). qRT-PCR and western blot were conducted to test mRNA expression and protein expression of SPN in ccRCC cells and normal cells, finding that mRNA expression and protein expression of SPN were considerably up-regulated in ccRCC cells than those in normal cells (Fig. 3d, e). We simulated that SPN might be a target gene of miR-129-5p in ccRCC cells, and it was verified to be up-regulated in ccRCC cells.

### miR-129-5p targeted binds to SPN

**Fig. 4:**
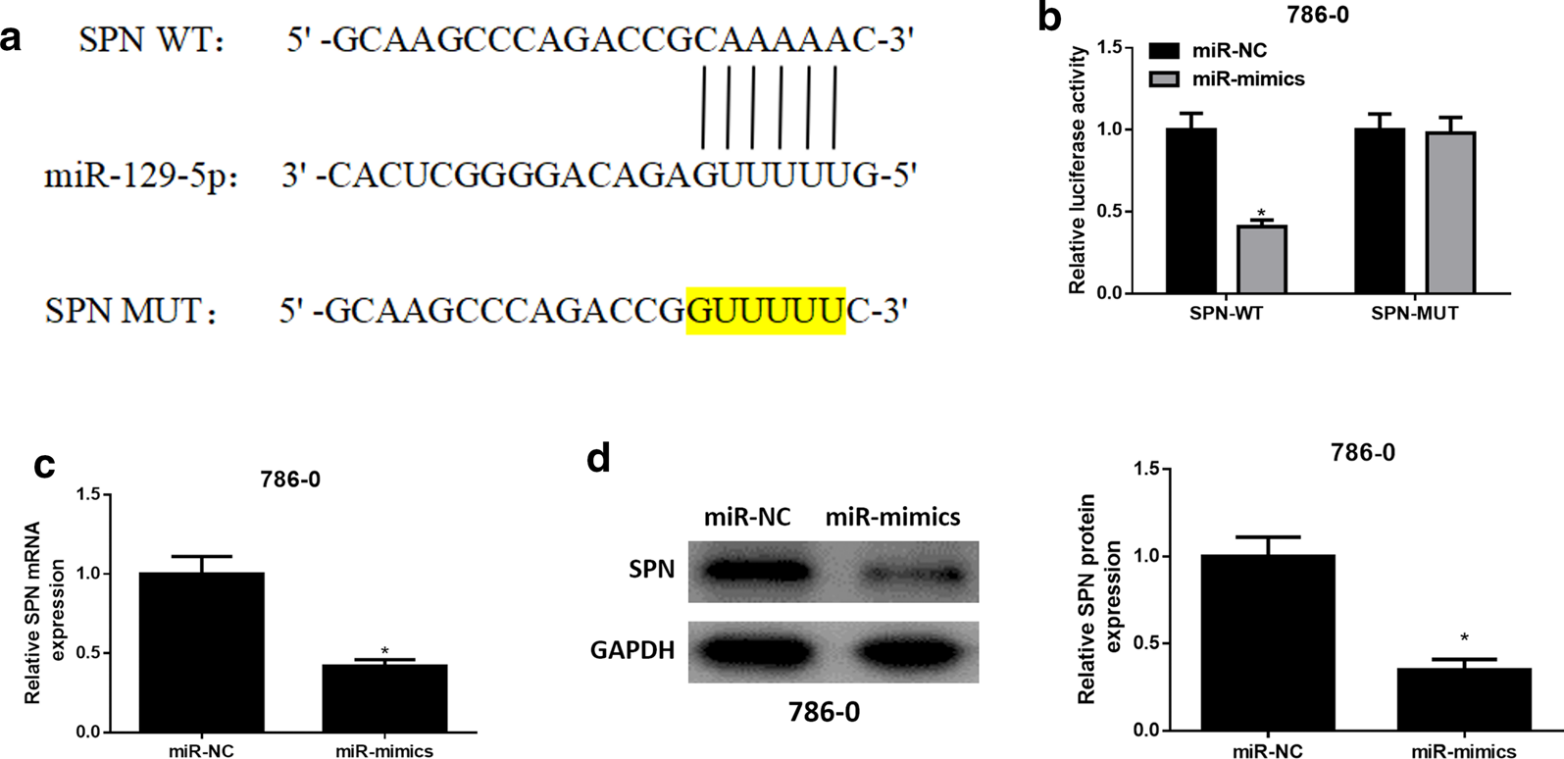
miR-129-5p targets and down-regulates SPN expression. **a** The binding sites of miR-129-5p on SPN 3’UTR were predicted by bioinformatics analysis; **b** The luciferase activity in different groups was detected by dual-luciferase assay; **c** mRNA expression and **d** protein expression of SPN in ccRCC cell line 786-O after miR-129-5p was overexpressed were measured by qRT-PCR and western blot; * *p* < 0.05

To further explore the regulatory effect of miR-129-5p on SPN, bioinformatics analysis was used to predict the binding sites of miR-129-5p on SPN 3’-UTR, indicating that there were binding sites between miR-129-5p and SPN (Fig. 4a). Subsequently, dual-luciferase assay validated the targeted binding relationship between miR-129-5p and SPN, finding that overexpression of miR-129-5p inhibited the luciferase activity of SPN-WT 3’-UTR, while had no influence on that of SPN-MUT 3’-UTR (Fig. 4b). qRT-PCR and western blot were performed to measure mRNA expression and protein expression of SPN, finding that mRNA expression and protein expression of SPN in ccRCC cell line 786-O were prominently decreased after miR-129-5p was overexpressed (Fig. 4c, d). Taken together, these findings elucidated that miR-129-5p targeted and down-regulated SPN in ccRCC cells.

### miR-129-5p targets SPN to regulate ccRCC cell malignant progression

**Fig. 5:**
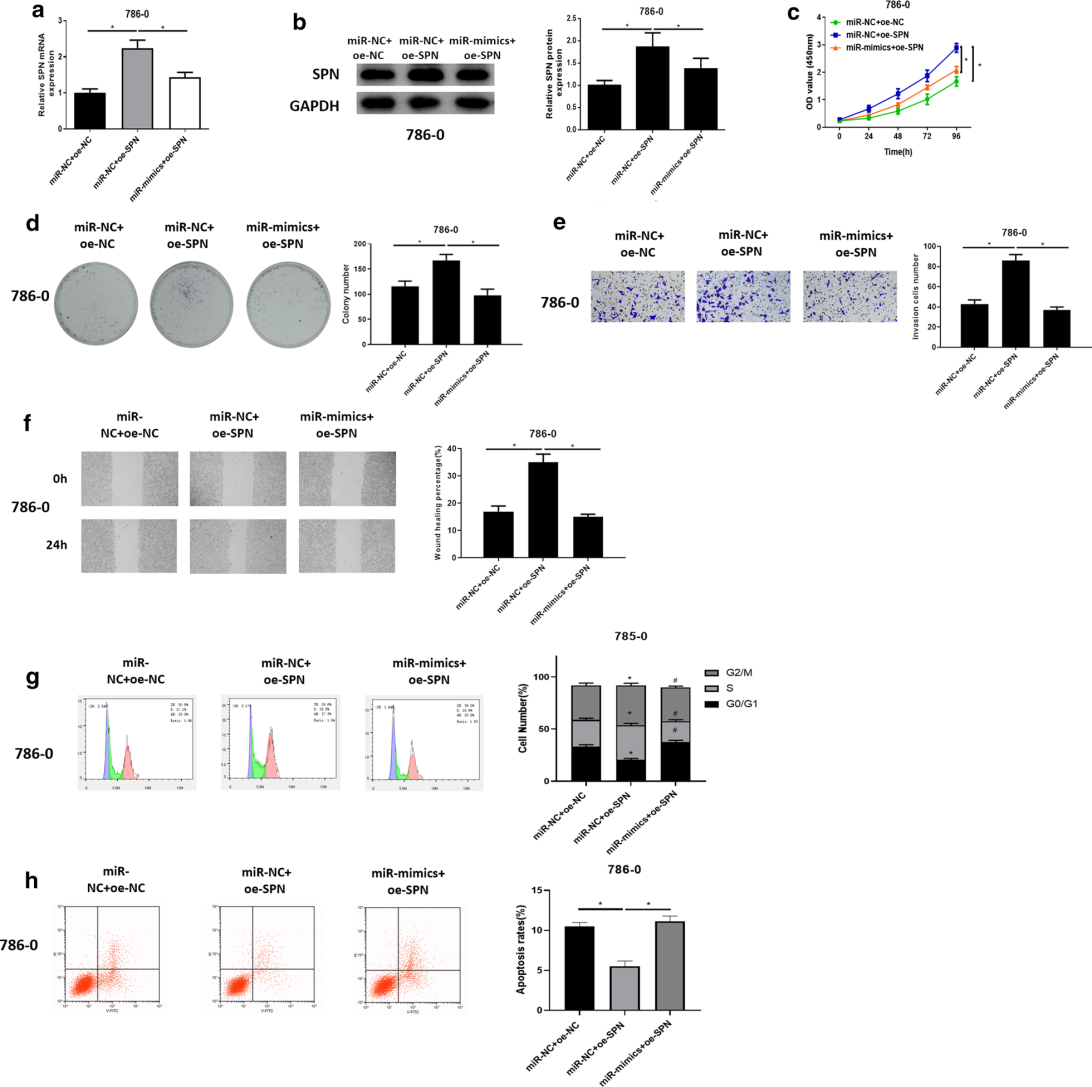
miR-129-5p targets SPN to regulate ccRCC cell malignant progression. **a** The mRNA expression and **b** protein expression of SPN in miR-NC + oe-NC, miR-NC + oe-SPN and miR-mimics + oe-SPN groups; **c** Cell proliferative ability, **d** colony forming ability, **e** cell invasive and **f** migratory abilities were detected by CCK-8 assay, colony formation assay, Transwell assay (100×) and wound healing assay (40×), respectively; **g** Cell cycle analyzed via flow cytometry; **h** Cell apoptosis evaluated by flow cytometry; * represents comparison with the miR-NC + oe-NC group, *p* < 0.05; # represents comparison with the miR-NC + oe-SPN group, *p* < 0.05

To investigate whether miR-129-5p regulates ccRCC cell behaviors by targeting SPN, we established miR-NC + oe-NC, miR-NC + oe-SPN and miR-mimics + oe-SPN groups. qRT-PCR and western blot were used to assess SPN expression in different groups, finding that SPN was potently up-regulated in miR-NC + oe-SPN group, whereas its expression was reversed in miR-mimics + oe-SPN group (Fig. 5a, b), which further illustrated that SPN was down-regulated by miR-129-5p. CCK-8 assay and colony formation assay suggested that overexpressing SPN markedly enhanced ccRCC cell proliferative ability, while overexpressing miR-129-5p significantly attenuated the promoting effect of SPN on ccRCC cells (Fig. 5c, d). Transwell assay and wound healing assay were employed to detect cell invasive and migratory abilities of ccRCC cells in different groups, finding that cell invasive and migratory abilities were remarkably increased after SPN was overexpressed, while such effect was reversed in miR-mimics + oe-SPN group (Fig. 5e, f). Similarly, experimental results of flow cytometry indicated that, compared with the NC group, the miR-NC + oe-SPN group showed more cells arrest in S and G2/M phases and decreased cell apoptosis. When miR-129-5p was elevated in the meantime, these effects were attenuated to a certain degree (Fig. 5g, h). Collectively, it could be seen that miR-129-5p suppressed cell proliferation, migration, invasion, induced cell cycle arrest and potentiated cell apoptosis via targeting SPN.

## Discussion

In recent years, accumulating studies have illustrated that miRNAs are key players in various cancers. Several miRNAs have been identified to participate in some important biological and pathological processes of ccRCC [15]. For instance, miR-182 [16], miR-30a [17] and miR-200c-3p [18] act as tumor suppressors to inhibit the progression of ccRCC by respectively targeting IGF1R, ADAM9 and SLC6A1, while miR-654 [19] and miR-543 [20] facilitate ccRCC cell proliferation and metastasis by targeting GK5 and Krüppel-like factor 6 (KLF-6), respectively. Generally speaking, miR-129-5p is considered to be a tumor suppressor and is down-regulated in prostate cancer (PC) [21], bladder cancer (BC) [22], etc. Wu Z et al. pointed out that the long non-coding RNA SNHG12 acts as a competing endogenous RNA to regulate MDM4 expression by competing with miR-129-5p in ccRCC [12]. In this study, we found that miR-129-5p was significantly down-regulated in ccRCC by bioinformatics analysis and cell experiments, and it was in agreement with the report of Wu Z et al.. Besides, miR-129-5p could act as a tumor suppressor to inhibit cell proliferation, migration, invasion, induce cell cycle arrest in G0/G1 phase and potentiate cell apoptosis. These findings help to understand the regulatory mechanism of miR-129-5p serving as a tumor suppressor in ccRCC, and also help to find a novel biomarker and targeted therapy for ccRCC.

Despite the fact that the regulatory mechanism of miR-129-5p in ccRCC has been reported, its molecular mechanism is still underway to exploit. On this basis, we investigated the regulatory mechanism of miR-129-5p in ccRCC, and further searched a novel potential therapeutic target for ccRCC. As indicated by bioinformatics analysis, SPN was observed to have the potential to be a target gene of miR-129-5p. SPN is capable of regulating intercellular adhesion, intracellular signal transduction, cell apoptosis, cell proliferation and metastasis[14]. There are few studies on the biological function of SPN in the progression of cancers, and only one study discussing the role of aberrant CD43 glycosylation as a cancer biomarker [23]. The effect of SPN on ccRCC and the regulatory mechanism of the miR-129-5p/SPN axis in ccRCC have not been reported to date. As revealed by cell experiments, we found that SPN was up-regulated in ccRCC cells, and after SPN was overexpressed, ccRCC cell proliferation, migration and invasion were increased, cell apoptosis was decreased, and cell cycle was found to arrest in S and G2/M phases, which suggested that high expression of SPN facilitated the malignant progression of ccRCC. Dual-luciferase assay further validated that there was a targeted binding relationship between miR-129-5p and SPN. Additionally, rescue experiments indicated that miR-129-5p suppressed ccRCC cell proliferation, migration and invasion by targeting SPN, promoted cell apoptosis and regulated cell cycle.

In sum, our study confirmed that miR-129-5p inhibits the progression of ccRCC by targeted down-regulating SPN, and first revealed the effect of the miR-129-5p/SPN axis on ccRCC cell proliferation, migration and invasion. These findings not only illustrate the regulatory mechanism of miR-129-5p and SPN in the progression of ccRCC, providing novel potential therapeutic targets for ccRCC, but also greatly deepen our knowledge about the effect of SPN on the progression of cancers and its upstream regulatory mechanism, bringing additional insight into the exploration of new biomarkers and target therapies for ccRCC.

## Supplementary Information

**Additional file 1.** Western blot picture of SPN.

**Additional file 2.** Normalized microRNA expression data of ccRCC patients in TCGA database.

**Additional file 3.** Normalized mRNA expression data of ccRCC patients in TCGA database.

## Data Availability

The data and materials in the current study are available from the corresponding author on reasonable request.
