# Supplementary figures and images for: miR-129-5p inhibits clear cell renal cell carcinoma cell proliferation, migration and invasion by targeting SPN
Source: Cancer Cell Int. 2021 May 17;21:263. doi: 10.1186/s12935-021-01820-3 (PMC8127191; doi:10.1186/s12935-021-01820-3)

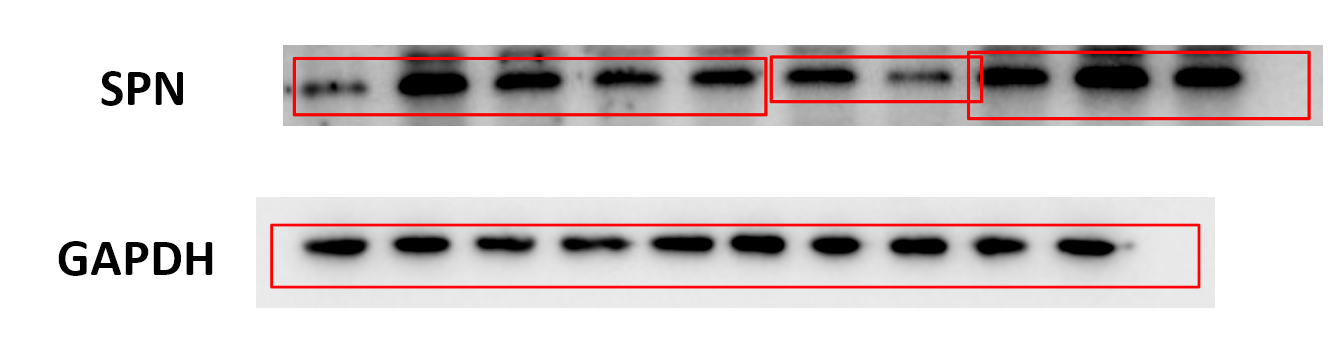

Supplement: Supplementary file 1 — Additional file 1. Western blot picture of SPN. [file 12935_2021_1820_MOESM1_ESM.tif]
